# The Road to Readiness: Commentary on the Journey of a Zoonotic H5 Influenza Vaccine Strain Update

**DOI:** 10.3390/vaccines14030203

**Published:** 2026-02-25

**Authors:** Alexander T. Kennedy, Ray Longstaff, James Fitzpatrick, Clare Hughes, Maria Teresa Malatesta, Raffaella Brandi, Joanne Beighton, Eve Versage, Nedzad Music, Howard Xu, Monica Pagni, Matthew Hohenboken, Beverly Taylor

**Affiliations:** 1 Seqirus UK Ltd., Maidenhead SL6 8AD, UK; alex.kennedy@seqirus.com (A.T.K.); ray.longstaff@seqirus.com (R.L.);; 2 Seqirus UK Ltd., Gaskill Road, Speke, Liverpool L24 9GR, UK; clare.hughes@seqirus.com (C.H.);; 3 Seqirus S.r.l., 53035 Siena, Italy; maria_teresa.malatesta@seqirus.com (M.T.M.); raffaella.brandi@seqirus.com (R.B.);; 4 Seqirus USA Inc., Waltham, MA 02451, USAnedzad.music@seqirus.com (N.M.);; 5 CSL Behring L.L.C., King of Prussia, PA 19406, USA

**Keywords:** influenza, pandemic, zoonotic vaccine, clade 2.3.3.4b, development, regulatory approval, pandemic preparedness

## Abstract

This commentary provides an overview of the development of a zoonotic influenza vaccine, in response to the emergence of an H5N1 subtype virus from clade 2.3.4.4b in mid-2020. When development was initiated, the World Health Organization (WHO) had recommended four candidate vaccine viruses; the A/Astrakhan/3212/2020-like strain was selected as it provided good coverage of circulating viruses and, critically, was available. To facilitate regulatory approval, the licence of an existing zoonotic vaccine, Aflunov (A/turkey/Turkey/01/2005)—a pre-pandemic monovalent A/H5N1 adjuvanted with MF59 and manufactured using the egg-based platform—was duplicated, with the plan to submit a variation to the duplicate licence for the strain update. This was supported by a ferret immunogenicity study using pseudoviruses (allowing the work to be conducted at a lower biosafety level), in conjunction with clinical data from the original Aflunov licence application, and a US study (NCT05874713) on another candidate zoonotic vaccine manufactured using the cell-based platform. Qualification batches for characterisation studies were manufactured at-risk, until calibrated, homologous reagents were available, and the final product release and stability studies were conducted, with rolling provision of stability data to health authorities. The vaccine was initially approved with a shorter shelf-life, allowing early distribution in certain countries, with later extension of the shelf-life once data became available. In terms of procurement and logistics, early consultation between the European Commission and EU member states resulted in the award of a Framework Contract for the initial supply of 665,000 doses to 15 states. Learnings from the development of this vaccine may help to improve pandemic readiness in the future.

## 1. Introduction 

Influenza A has caused four pandemics since the 20th century [1,2] and remains one of the most likely viruses to emerge as the cause of a respiratory pandemic [3,4]. Among the influenza viruses of pandemic potential (IVPP), the highly pathogenic avian influenza (HPAI) H5 subtype is of significant concern [5]. In mid-2020, an HPAI H5N1 subtype virus from clade 2.3.4.4b emerged in central Europe from reassortment between an HPAI H5N8 subtype and local low pathogenic avian influenza (LPAI) viruses [6] and has raised concerns about its potential to cause a pandemic. Since its emergence, HPAI H5N1 has infected millions of wild birds and domestic poultry, spreading across Europe, Asia, North America, and South America [7]. Of particular concern is its capacity to infect mammals, suspected cases of mammal-to-mammal transmission, and the recurrent detection of mutations, such as PB2 E627K, associated with mammalian adaptation [7]. More than 70 sporadic human infections have been reported in connection with the outbreak, the majority of which have been mild and associated with exposure to infected dairy cattle in the USA [8,9,10,11].

As the influenza virus is a known pandemic threat, plans for public health and social measures, as well as medical countermeasures, have been developed to prepare for and protect against any emerging pandemic. Public health and social measures include the use of personal protective equipment [12] and social distancing [13], while the available medical countermeasures are antivirals [14] and both zoonotic (aka pre-pandemic) vaccines and pandemic vaccines for use in humans. For pandemic influenza vaccines, a core dossier is submitted and approved under exceptional circumstances by health authorities during the interpandemic period. The dossier, which is a ‘mock-up’ vaccine containing an IVPP strain under European regulatory procedures, is updated in the event of a pandemic to closely match the pandemic strain and facilitate rapid approval [15]. In contrast, zoonotic influenza vaccines are fully authorised by health authorities, allowing them to be used immediately in response to an outbreak or early in an emerging pandemic, prior to availability of an updated pandemic vaccine based on the pandemic virus [16]. Zoonotic vaccines are typically formulated with a strain of the virus isolated from animals or from a zoonotic human case [16].

To ensure manufacturers have access to relevant viruses that provide good coverage of the currently circulating viruses for inclusion in vaccines, the World Health Organization (WHO) conducts surveillance of both seasonal influenza viruses and IVPPs throughout the year. They do this via a network of National Influenza Centres (NIC) and WHO collaborating centres (CCs), which are part of a Global Influenza Surveillance and Response system (GISRS) [17]. As novel viruses are identified, they are compared with existing reference viruses from the same clade to check that they are immunologically similar. Based on the test results, GISRS determines whether a new candidate vaccine virus (CVV) is needed. If so, this recommendation is presented at the biannual WHO vaccine composition meetings [18]. 

For the HPAI H5 subtype, a total of 51 CVVs are listed as available or pending [19], and development work, stopping short of production of a finished vaccine, has been undertaken for many of these as part of good pandemic preparedness. However, licenced zoonotic vaccines have typically been registered as containing CVV strains from the 2000s [20,21,22,23]. The European Medicines Agency (EMA) has pathways to apply for a strain update to these zoonotic vaccines [16]; however, these require submission of data generated during commercial-scale production of the new, strain-updated, candidate vaccine [16].

There are limited opportunities for the production of zoonotic vaccines, including the batches needed for generation of data to support a strain update, because manufacturers conduct seasonal influenza vaccine-manufacturing campaigns twice a year, with one for each hemisphere [16]. The timing of these campaigns is pre-determined for each season and critical to ensuring that vaccines are available in time to protect populations from seasonal influenza epidemics. In the event of a pandemic emergency being declared, production of seasonal vaccines would cease, and the entire “warm” manufacturing base would transition to the rapid production of pandemic influenza vaccines [16]. During the 2009 H1N1 pandemic, the transition from seasonal to pandemic influenza vaccines led to registration of the pandemic vaccine approximately 100 days after the WHO’s declaration of the pandemic [4]. The challenge during interpandemic periods is that there are only brief windows of time available between seasonal campaigns for the manufacture of zoonotic influenza vaccines. These windows are further constrained to accommodate routine maintenance and testing of facilities and equipment. 

Due to the complexity involved, the manufacturing of zoonotic vaccines requires careful consideration and planning, alongside clear demand for vaccine from governments. Timely coordination and alignment between public health authorities, regulators, and payors is essential to make the best use of the brief production windows. In addition, the necessary CVVs and reagents must be available, and while CVV acquisition and seed preparation can be done in advance, all other activities, including large-scale production of monovalent vaccine antigen, adjuvant manufacture, formulation, filling, packing, and other related regulatory and quality tasks, must be scheduled within these windows [24].

To date, CSL Seqirus holds the only authorised zoonotic (pre-pandemic) influenza vaccines targeting H5 strains in Europe [25]. With respect to pandemic preparedness vaccines in Europe that can be rapidly modified to protect people in a pandemic situation, only CSL Seqirus, GlaxoSmithKline and AstraZeneca have authorised vaccines at this time. Registrations in other regions may vary. For example, in the United States, CSL Seqirus, Sanofi and GlaxoSmithKline all have approved H5-containing vaccines [26,27,28]. CSL Seqirus has extensive experience in the field of pandemic and zoonotic influenza vaccines, as evidenced by responding to the 2009 H1N1 pandemic (as its predecessor organisations) with vaccines registered in the US and Europe 97 to 111 days after the pandemic declaration and long-term partnerships with agencies such as the United States Biomedical Advanced Research and Development Authority (BARDA). CSL Seqirus has well-established egg-based and cell-based platforms of manufacture, with both having associated authorised zoonotic influenza and pandemic influenza preparedness vaccines. In addition, CSL Seqirus has extensive experience with the use of MF59 adjuvant, which has been key in developing effective and dose-sparing vaccines for potential pandemic scenarios.

**Figure 1:**
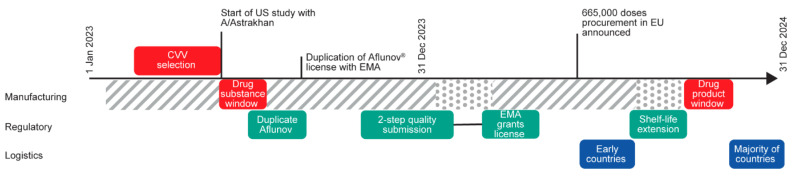
CSL Seqirus timeline for the regulatory approval and manufacture of a H5 zoonotic vaccine containing a clade 2.3.4.4b CVV. Hashed and dotted lines are to represent periods when manufacturing is not possible due to use for seasonal manufacture. CVV, candidate vaccine virus; EMA, European Medicines Agency; EU, European Union; US, United States.

**Figure 2:**
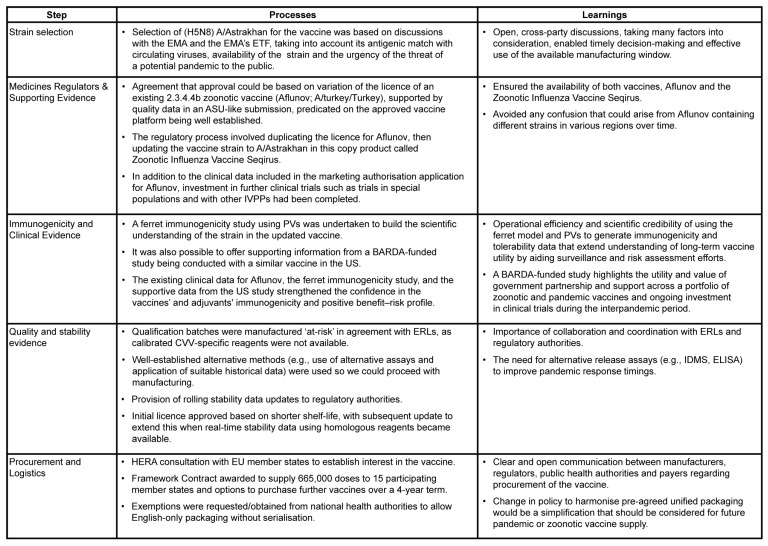
Key learnings from the development of a H5 zoonotic clade 2.3.4.4b vaccine. ASU, Annual Strain Update; BARDA, Biomedical Advanced Research and Development Authority; CVV, candidate vaccine virus; ELISA, Enzyme-Linked Immunosorbent Assay; EMA, European Medicines Agency; ERL, Essential Regulatory Laboratories; ETF, Emergency Task Force; EU, European Union; HERA, Health Emergency Preparedness and Response Authority; IDMS, Isotope Dilution Mass Spectrometry; IVPP, influenza viruses with pandemic potential; PV, pseudovirus.

To address the clear public health need for a zoonotic vaccine relevant to circulating virus strains, CSL Seqirus navigated these challenges and initiated activity to produce and register an MF59-adjuvanted H5 zoonotic vaccine containing a clade 2.3.4.4b CVV, manufactured using an egg-based platform. A duplicate marketing authorisation licence was sought from the EMA for Aflunov (CSL Seqirus) [20], an already EMA-licenced zoonotic vaccine registered with H5N1 A/turkey/Turkey/01/2005 from clade 2.2.1, and a variation was later submitted to update the strain in the new licence [29]. In this article, we describe our experience in developing this vaccine (Figure 1) and present our experiences and lessons learned (Figure 2) with the aim of improving pandemic and outbreak readiness in the future.

## 2. Strain Selection

**Figure 3:**
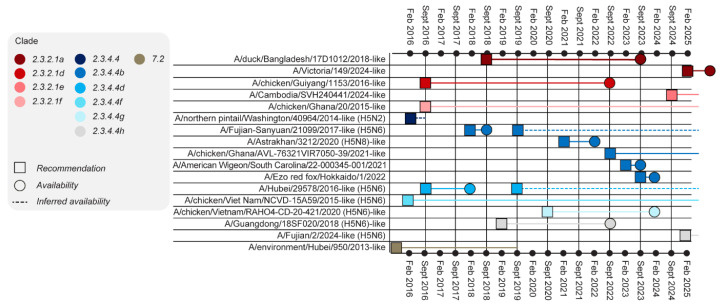
Timeline from recommendation to availability of WHO-recommended H5 CVVs. Only CVVs first recommended from the 2015 September report onward are shown here. Some rows contain multiple recommendation boxes; these represent strains that were initially recommended and became available but were then reverted. In the event a CVV was discontinued before it became available the open-ended line terminates on the date it was removed. CVV, candidate vaccine virus; WHO, World Health Organization.

In the event of a pandemic emergency, as defined by the WHO International Health Regulations (IHR), CVVs for the emerging influenza pandemic virus would be produced quickly [27]. In contrast, during interpandemic periods, WHO and GISRS prioritise CVVs for seasonal influenza vaccines to ensure timely availability for the upcoming season, with recommendations updated twice yearly for zoonotic influenza CVVs [30]. CVVs for IVPPs, which pose no immediate pandemic threat, may take months or years to prepare (Figure 3) due to a number of reasons. The WHO Pandemic Influenza Preparedness (PIP) framework facilitates access to IVPPs [31], but some viruses are governed by the Nagoya Protocol of the Convention on Biological Diversity (CBD) [32], which can result in further delays in accessing them to develop CVVs.

A key decision for influenza vaccine development is the selection of a strain(s) that match(es) those that are circulating. Choosing the most appropriate strain for zoonotic vaccines is challenging, as it involves a delicate balance between the likelihood of which CVV (and its related potency reagents) will be available in time to meet the production timeline, the risk of missing an opportunity to respond to an outbreak, and the public health threat. 

Consultations with the EMA and the EMA’s Emergency Task Force (ETF) regarding the update of the zoonotic vaccine strain to address the threat of the new clade 2.3.4.4b took place between March and May 2023. At that time, the WHO had recommended four clade 2.3.4.4b CVVs: (H5N8) A/Astrakhan/3212/2020 (A/Astrakhan), (H5N6) A/Fujian-Sanyuan/21099/2017-like (A/Fujian-Sanyuan), (H5N1) A/chicken/Ghana/AVL-763_21VIR7050-39/2021-like (A/chicken/Ghana), and (H5N1) A/American wigeon/South Carolina/22-000345-001/2021) (A/American wigeon) [33,34,35]. Post-infection ferret antisera data indicated that the A/Astrakhan and A/American wigeon CVVs were suitable candidates with good coverage of circulating strains in clade 2.3.4.4b (A/Astrakhan/3212/2020-like, A/American wigeon/South Carolina/22-000345-001/2021-like) [33,36]; however, only the A/Astrakhan CVV was available at the time of consultations (with the A/American wigeon only becoming available in September 2023 [37]). The (H5N8) A/Astrakhan CVV was selected as the timing of the CVV availability was critical to meeting the manufacturing window, and the haemagglutinin (HA) protein sequences of the A/Astrakhan and A/American Wigeon viruses were very similar at the amino acid level [33,38]. In addition, haemagglutination inhibition (HAI) assays show no evidence that antigenicity of the H5 component is affected by which NA type it is paired with (personal correspondence with WHO CC). Therefore, the decision to select the (H5N8) A/Astrakhan CVV was based not only on its antigenic match with circulating viruses, but also on practical considerations such as availability of CVVs, public health urgency, and timing pressures.

There are risks associated with selecting strains before a CVV is available. This is illustrated by the fact that the A/chicken/Ghana and A/Fujian-Sanyuan CVVs (which were selected as CVVs in September 2022 and February 2018, respectively) remain pending according to the February 2025 WHO report [37,39] (Figure 3). The option of awaiting new CVVs was considered in consultation with regulators; however, public health concerns grew as clade 2.3.4.4b became the dominant strain causing outbreaks in birds and mammals across Europe and the Americas. From 2021 to 2023, outbreaks included those in large marine mammals in South America and on fur farms in Finland, and there was suspected mink-to-mink transmission in Spain [40,41,42,43,44], as well as sporadic human cases. In addition, the timeline for the availability of a new CVV remained unclear, increasing the risk of missing the designated manufacturing window. In light of these considerations, A/Astrakhan was selected as the strain. This decision was approved by both the EMA and ETF and it was a result of open, cross-party discussions that took many factors into consideration, enabling a timely decision and effective use of the available manufacturing window [38].

## 3. Medicines Regulators and Supporting Evidence 

For seasonal influenza vaccines, Annual Strain Update (ASU) processes are well established and allow vaccines to be updated with the changes in strain composition recommended by the WHO [45], based on quality (chemistry and manufacturing) data for the update strains. The EMA also applies this guideline to zoonotic strain changes [16], but this process is less well established than for seasonal vaccines.

To update the existing approved zoonotic vaccine to a clade 2.3.4.4b strain, two crucial regulatory agreements were obtained. Firstly, in consultation with the EMA and the ETF, the A/Astrakhan CVV was accepted as the vaccine strain, as discussed in Section 2. Secondly, the agreed update was based on quality (chemistry and manufacturing) data for the update strains in an ASU-like submission, predicated on the EMA-approved vaccine platform being well established. The regulatory process involved duplicating the existing licence of the zoonotic vaccine Aflunov (A/turkey/Turkey) and updating the vaccine strain to A/Astrakhan in a copy product called Zoonotic Influenza Vaccine Seqirus. This process ensured the availability of both vaccines—the original with the A/turkey/Turkey strain and the updated one with the A/Astrakhan strain—and avoided any confusion that could arise from Aflunov containing different strains in various regions over time [38].

## 4. Immunogenicity and Clinical Evidence

A key element of the response to clade 2.3.4.4b was having a licenced zoonotic vaccine that could be duplicated and updated with a relevant CVV. To have this in place and able to support a strain update required that, during an interpandemic period, the necessary clinical trials and other components needed to obtain licensure of a zoonotic vaccine had been completed. In addition to the clinical data included in the marketing authorisation application submitted for Aflunov, investment in further clinical trials such as trials in special populations and with other IVPPs had already been completed [20,46,47,48]. This continued expansion of the clinical database further strengthened the confidence in immunogenicity of the vaccines and adjuvants, as well as the positive benefit–risk profile. 

A study funded by the US Government investigating the immunogenicity and safety profile of an A/Astrakhan vaccine (NCT06560151), which contains the same amount of antigen and MF59 adjuvant as the proposed updated H5 zoonotic vaccine but manufactured on a different platform (i.e., cell-based), was also underway at this time [49,50]. Like Aflunov, the vaccine in question was also licenced with a regulator, the US Food and Drug Administration (FDA), using the A/turkey/Turkey strain [26]. It was acknowledged that lessons learned from the US-based trial, albeit with a vaccine manufactured on a cell-based platform, would be relevant to confirming the hypothesis that a similar level of immune response to vaccines that contained A/turkey/Turkey would be seen in a vaccine that contained the A/Astrakhan strain. With the agreement of the US Government, the interim study report from the A/Astrakhan study was shared with the EMA and ETF. The Biomedical Advanced Research and Development Authority (BARDA)-funded study in the US with the A/Astrakhan strain [46,48] highlights the utility and value of a government partnership across a portfolio of zoonotic and pandemic vaccines, and ongoing investment in clinical trials during the interpandemic period.

Although not a requirement, it was agreed with the EMA and ETF that, in addition to the cell-based A/Astrakhan clinical trial data that was voluntarily shared with them, an immunogenicity study in ferrets would be undertaken to build the scientific understanding of the strain-updated vaccine. Ferrets are widely recognised as the most appropriate small-animal model for the study of influenza virus infection and vaccination [51]. This model has been essential in identifying immune markers of protection during vaccine evaluation [52].

HAI antibody titres are currently accepted as a statistical correlate of protection (CoP) against influenza in humans, supported by numerous studies dating back to the 1970s [53,54,55,56,57]. A similar relationship between HAI titres and protection has been reported in ferrets [58], justifying the use of immunogenicity data alone when HAI responses are adequate. Previous studies have demonstrated that ferret immunogenicity data align well with observed vaccine performance in humans, allowing extrapolation from animal to clinical settings [59]. Regulatory agencies including EMA and the US FDA explicitly recognise the ferret as the preferred non-clinical model for evaluating immunogenicity, protective efficacy and safety in pandemic influenza vaccine development [60,61,62]. 

The immunogenicity study involved immunising ferrets with one or two doses of the MF59-adjuvanted vaccine, containing 12.5 µg HA of the H5N8 A/Astrakhan antigen, followed by serum collection at several time points. The study design was tailored to mirror practical field-use scenarios: A prime-only schedule versus a prime-boost strategy. Immunogenicity was assessed using both HAI and neutralisation assays against a panel of pseudoviruses (PVs) representing homologous (A/Astrakhan) and heterologous H5Nx viruses (e.g., A/American wigeon/; A/turkey/Turkey). As expected, the study results supported the tolerability of the A/Astrakhan vaccine and its immunogenicity against both the vaccine strain and closely related heterologous strains [63] Another key takeaway of the study was the value of using PVs, which showed good correlation to live virus results. PVs allow this type of work to be conducted at a lower biosafety level than required for live viruses, reducing cost and complexity, and making such studies faster and easier to initiate [64].

The ferret sera generated in this immunogenicity study have offered scientific value beyond their initial purpose, and these subsequent analyses have also benefitted from the simplicity afforded by using PVs. These samples have been used to assess immunological cross-reactivity to newly emerging H5Nx viruses, including isolates associated with the ongoing H5N1 2.3.4.4b outbreaks in cattle in the US. These analyses have provided timely insights into vaccine coverage against circulating strains and also provide evidence of the potential increased breadth of response afforded by the MF59-adjuvant [63]. Overall, these results have informed pandemic and outbreak preparedness and response activities.

Subsequently, Finland has since conducted a vaccination programme, through which a human clinical study undertaken by Liedes and colleagues found the vaccine to be immunogenic with cross-reactive antibodies to clade 2.3.4.4b viruses from Europe and North America [65], confirming the accuracy of these ferret results.

## 5. Quality and Stability Evidence

Following selection of the A/Astrakhan strain, three qualification batches of the vaccine were manufactured to perform characterisation studies and to generate release and stability data to support the strain update application. This had to be carried out ‘at-risk’ since calibrated CVV-specific reagents required to measure potency at release and for ongoing stability were not available from the Essential Regulatory Laboratories (ERLs). The use of alternative assays and application of suitable historical data from our manufacturing experience has been used in the past in this situation for both seasonal and zoonotic influenza vaccine production where, because of the timing for any new strains selected, manufacturing starts on the assumption reagents will be available when needed [66]. 

Heterologous reagents were available for another CVV from clade 2.3.4.4b; however, after discussion with health regulators, it was determined that these were not acceptable for use with the selected strain. Due to the continuous risk posed by ongoing outbreaks and the need to meet supply timelines for interested governments, we proceeded with drug substance and drug product manufacturing at-risk in the absence of homologous reagents, using well-established alternative methods in order to mitigate delays.

These challenges with reagents were managed through collaboration and coordination with the ERLs. Once the homologous reagents were available, we immediately progressed to final product release and commencement of stability studies. In contrast, during a declared pandemic, production of reagents would need to be prioritised, and health authorities may accept the use of heterologous reagents or other alternatives, depending on the urgency of the situation. In agreement with the regulatory agencies, we provided rolling stability data once the calibrated reagents had been received. A shorter shelf-life was initially approved for the vaccine, and this was extended as additional real-time stability data became available.

Delivery of the vaccine to many countries was deferred until this longer shelf-life was approved, although some countries did take on shorter shelf-life stock to facilitate vaccine programmes [67]. In a pandemic setting, vaccine shelf-life may be of lower concern to health authorities as vaccination processes are streamlined, and the demand and uptake are likely to be higher with the vaccine being utilised in a shorter timeframe (most likely deployed as quickly as it is received). As seen for CVVs, achieving optimal prioritisation of seasonal influenza and IVPP reagents is important to enable release and stability testing of the vaccine. Antigen production routinely starts months before reagent availability; however, vaccines cannot be released and deployed until calibrated homologous reagents are available. It is estimated by the WHO ERLs that reagent production and calibration take at least 12 weeks to complete [68], and this needs to be considered in pandemic response planning. Alternative release assays that do not rely on generating strain-specific sheep antisera could also improve pandemic response timings. Several such assays have been under evaluation for a number of years (e.g., IDMS, ELISA) [69].

## 6. Procurement and Logistics

The timing and production of zoonotic vaccines involves a number of risks, as key steps including procurement of materials, manufacture of drug substance and drug product, conducting testing and generating quality data, and preparing and submitting updates to regulatory dossiers must be undertaken. As such, clear and open communication between manufacturers, regulators, public health authorities, and payors regarding procurement of the vaccine is critical. The COVID-19 pandemic demonstrated how government orders are a powerful tool to alleviate financial risk [70]. The commitment of several government health authorities played an important role in supporting the initiation of manufacturing activities for the zoonotic vaccine. Notably, the Health Emergency Preparedness and Response Authority (HERA) in the European Commission led a consultation with EU member states to establish interest in the vaccine, which confirmed demand in the region and initiated a joint procurement procedure. This ultimately resulted in the awarding of a Framework Contract to supply 665,000 doses to 15 participating member states and options to purchase further vaccines over a 4-year term. Several other countries, including the United Kingdom, have also procured the A/Astrakhan vaccine [71].

Supplying vaccines rapidly across multiple countries requires flexibility and efficiency, but country-specific packaging increases complexity, slows down distribution and, determining when to order packaging for specific countries, adds risk. In this case, most parties agreed that supplying vaccines in English-only packaging without serialisation would simplify matters. However, existing practices required all parties to obtain exemptions from national regulatory agencies, which delayed delivery.

After the EMA approval of the shelf-life extension in September 2024 [72] (Figure 1), vaccines to fulfil the European HERA order were formulated, filled into syringes, labelled, and packaged for distribution. Distribution was undertaken as quickly as possible while managing the variability in order volumes from different countries, setting up supply routes, and applying for exemptions from each national regulatory agency for single-language packaging. Other CSL Seqirus-manufacturing sites in Australia and the USA also responded to produce clade 2.3.4.4b zoonotic vaccine doses. To date, across the three manufacturing sites, 20 governments have been supplied with zoonotic clade 2.3.4.4b vaccines [73]. Based on publicly available information, the Canadian and Japanese governments have also acquired stockpiles from manufacturers with local facilities. 

## 7. Conclusions

Maintaining influenza pandemic readiness is complex and involves numerous steps to ensure a swift response to both pandemics and outbreaks. By holding pre-pandemic zoonotic vaccines, either as bulk of antigen or as finished vaccines, governments can respond rapidly to protect front-line workers during outbreaks of zoonotic influenza.

Timely detection of emerging zoonotic influenza viruses, including HPAI, and rapid and open sharing of the viruses and their genetic information are critical. It is also important to have the zoonotic CVVs available from the WHO collaborating centres and reassorting laboratories as soon as possible, given the brief windows of opportunity available between seasonal campaigns, so they can be supplied to manufacturers to add to the library of pre-pandemic viruses and to facilitate preliminary characterisation work.

The H5N8 A/Astrakhan CVV was selected to access the earliest possible manufacturing window between seasonal campaigns, responding to increased demand from government health authorities and to avoid a delay of at least 6 months until the next available window. The resulting pre-pandemic zoonotic vaccine, containing a clade 2.3.4.4b strain, initially a duplicate licence registered by the Medicines and Healthcare products Regulatory Agency (MHRA) in October 2023, was followed by a strain update in March 2024 and by the EMA in April 2024.

This vaccine was supported by data from the ferret model and the use of PVs to generate immunogenicity and tolerability data. The experience gained through this approach underscores the value of non-clinical models alone for zoonotic vaccines. It also demonstrates the operational efficiency and scientific credibility of using the ferret model and PVs to generate immunogenicity and tolerability data that extend understanding of long-term vaccine utility by aiding surveillance and risk-assessment efforts [63]. Additionally, the ferret study and results of the Finnish clinical study, although not pre-requisites, confirm that the strain update based on quality data produced the expected outcomes. Another lesson from this process was that pre-agreed, unified packaging is a simplification that should be considered for future pandemic or zoonotic vaccine supply. It reduces risks, making it easier to justify holding labelled rather than naked stock, gives flexibility in allocation, enables more equitable supply options by avoiding the need to sequentially swap between labels during packing, and shares the cost of labels and cartons. Ultimately, this can lead to improvements in supply times and potential re-allocation of stock between countries themselves.

This experience highlights the critical role of open, multi-stakeholder discussion and collaboration, the importance of taking decisive action within limited timelines, as well as integrating scientific rigour with practical application in response to outbreaks. It also underscores the necessity for ongoing coordination with international surveillance systems, such as GISRS, and considerations in balancing seasonal and IVPP CVV development and reagent supply. Collectively, these approaches can support timely and feasible vaccine updates in response to zoonotic threats and outbreaks as they arise.

## Data Availability

No new data were analysed in this study. Data sharing is not applicable to this article.

## Abbreviations

ASU	Annual Strain Update
CBD	Convention on Biological Diversity
CC	collaborating centre
CoP	correlate of protection
CVV	candidate vaccine virus
ELISA	Enzyme-Linked Immunosorbent Assay
EMA	European Medicines Agency
ERL	Essential Regulatory Laboratory
ETF	Emergency Task Force
FDA	Food and Drug Administration
GISRS	Global Influenza Surveillance and Response system
HA	haemagglutinin
HAI	haemagglutination inhibition
HERA	Health Emergency Preparedness and Response Authority
HPAI	highly pathogenic avian influenza
IDMS	Isotope Dilution Mass Spectrometry
IHR	International Health Regulations
IVPP	influenza viruses of pandemic potential
LPAI	low pathogenic avian influenza
MHRA	Medicines and Healthcare products Regulatory Agency
NIC	National Influenza Centres
PIP	Pandemic Influenza Preparedness
PV	pseudovirus
WHO	World Health Organization

## References

[1] Girard M.P., Tam J.S., Assossou O.M., Kieny M.P. (2010). The 2009 A (H1N1) influenza virus pandemic: A review. Vaccine.

[2] Kilbourne E.D. (2006). Influenza pandemics of the 20th century. Emerg. Infect. Dis..

[3] Morens D.M., Park J., Taubenberger J.K. (2023). Many potential pathways to future pandemic influenza. Sci. Transl. Med..

[4] Rockman S., Taylor B., McCauley J.W., Barr I.G., Longstaff R., Bahra R. (2022). Global Pandemic Preparedness: Optimizing Our Capabilities and the Influenza Experience. Vaccines.

[5] Kuiken T., Fouchier R.A.M., Koopmans M.P.G. (2023). Being ready for the next influenza pandemic?. Lancet Infect. Dis..

[6] World Health Organization (2024). Joint FAO/WHO/WOAH Preliminary Assessment of Recent Influenza A(H5N1) Viruses. https://www.who.int/publications/m/item/joint-fao-who-woah-preliminary-assessment-of-recent-influenza-a(h5n1)-viruses.

[7] Xie Z., Yang J., Jiao W., Li X., Iqbal M., Liao M., Dai M. (2025). Clade 2.3.4.4b highly pathogenic avian influenza H5N1 viruses: Knowns, unknowns, and challenges. J. Virol..

[8] Chrzastek K., Lieber C.M., Plemper R.K. (2025). H5N1 Clade 2.3.4.4b: Evolution, Global Spread, and Host Range Expansion. Pathogens.

[9] Hermann E., Krammer F. (2025). Clade 2.3.4.4b H5N1 neuraminidase has a long stalk, which is in contrast to most highly pathogenic H5N1 viruses circulating between 2002 and 2020. mBio.

[10] Pan American Health Organization (2025). Epidemiological Update Avian Influenza A(H5N1) in the Americas Region. https://www.paho.org/sites/default/files/2025-03/2025-mar-4-phe-epidupdate-avianinfluenza-eng-final.pdf.

[11] Webby R.J., Uyeki T.M. (2024). An Update on Highly Pathogenic Avian Influenza A(H5N1) Virus, Clade 2.3.4.4b. J. Infect. Dis..

[12] Xiao J., Shiu E.Y.C., Gao H., Wong J.Y., Fong M.W., Ryu S., Cowling B.J. (2020). Nonpharmaceutical Measures for Pandemic Influenza in Nonhealthcare Settings—Personal Protective and Environmental Measures. Emerg. Infect. Dis..

[13] Fong M.W., Gao H., Wong J.Y., Xiao J., Shiu E.Y., Ryu S., Cowling B.J. (2020). Nonpharmaceutical Measures for Pandemic Influenza in Nonhealthcare Settings—Social Distancing Measures. Emerg. Infect. Dis..

[14] Bright R.A. (2025). Optimizing Antiviral Stockpiles for Pandemic Response: A Strategic Framework. J. Infect. Dis..

[15] European Medicines Agency (2008). Guideline On Dossier Structure and Content for Pandemic Influenza Vaccine Marketing Authorisation Application (Revision). https://www.ema.europa.eu/en/documents/scientific-guideline/guideline-dossier-structure-and-content-pandemic-influenza-vaccine-marketing-authorisation-application-revision-superseded_en.pdf.

[16] European Medicines Agency (2023). Influenza Vaccines—Submission and Procedural Requirements—Scientific Guideline. https://www.ema.europa.eu/en/documents/scientific-guideline/guideline-influenza-vaccines-submission-and-procedural-requirements-rev2_en.pdf.

[17] World Health Organization (2025). Global Influenza Surveillance and Response System (GISRS). https://www.who.int/initiatives/global-influenza-surveillance-and-response-system.

[18] World Health Organization (2024). Recommendations Announced for Influenza Vaccine Composition for the 2024–2025 Northern Hemisphere Influenza Season. https://www.who.int/news/item/23-02-2024-recommendations-announced-for-influenza-vaccine-composition-for-the-2024-2025-northern-hemisphere-influenza-season.

[19] World Health Organization (2025). Genetic and Antigenic Characteristics of Zoonotic Influenza a Viruses and Development of Candidate Vaccine Viruses for Pandemic Preparedness. https://cdn.who.int/media/docs/default-source/influenza/who-influenza-recommendations/vcm-sh-2025/c.-26-september-2025-antigenic-and-genetic-characteristics-of-zoonotic-influenza-a-viruses-and-development-of-candidate-vaccine-viruses-for-pandemic-preparedness.pdf.

[20] European Medicines Agency (2024). Aflunov Summary of Product Characteristics. https://www.ema.europa.eu/en/documents/product-information/aflunov-epar-product-information_en.pdf.

[21] European Medicines Agency (2025). Incellipan Summary of Product Characteristics. https://www.ema.europa.eu/en/documents/product-information/incellipan-epar-product-information_en.pdf.

[22] European Medicines Agency (2022). Adjupanrix Summary of Product Characteristics. https://www.ema.europa.eu/en/documents/product-information/adjupanrix-epar-product-information_en.pdf.

[23] European Medicines Agency (2025). Foclivia Summary of Product Characteristics. https://www.ema.europa.eu/en/documents/product-information/foclivia-epar-product-information_en.pdf.

[24] European Medicines Agency (2024). Assessment Report: Celldemic. https://www.ema.europa.eu/en/documents/assessment-report/celldemic-epar-public-assessment-report_en.pdf.

[25] European Medicines Agency (2026). Zoonotic Influenza Vaccine Seqirus—Authorization. https://www.ema.europa.eu/en/medicines/human/EPAR/zoonotic-influenza-vaccine-seqirus#authorisation-details.

[26] Food and Drug Administration (2023). Audenz Prescribing Information. https://www.fda.gov/files/vaccines%2C%20blood%20%26%20biologics/published/Package-Insert-AUDENZ_2.pdf.

[27] GlaxoSmithKline (2013). H5N1 Vaccine Approved by the U.S. FDA as Pandemic Influenza Preparedness Measure. https://www.gsk.com/en-gb/media/press-releases/h5n1-vaccine-approved-by-the-us-fda-as-pandemic-influenza-preparedness-measure.

[28] U.S. Department of Health and Human Services Food and Drug Administration (2007). Sanofi Influenza Virus Vaccine—US FDA Prescribing Information. https://www.fda.gov/media/74534/download.

[29] European Medicines Agency (2024). Type II Variation Assessment Report. https://www.ema.europa.eu/en/documents/variation-report/zoonotic-influenza-vaccine-seqirus-h-c-006375-ii-0001-epar-assessment-report-variation_en.pdf.

[30] World Health Organization (2024). Recommended Composition of Influenza Virus Vaccines for Use in the 2024–2025 Northern Hemisphere Influenza Season. https://www.who.int/publications/m/item/recommended-composition-of-influenza-virus-vaccines-for-use-in-the-2024-2025-northern-hemisphere-influenza-season.

[31] World Health Organization (2021). Pandemic Influenza Preparedness Framework for the Sharing of Influenza Viruses and Access to Vaccines and Other Benefits. https://iris.who.int/server/api/core/bitstreams/4e22d25c-7084-4832-a0ff-667eac7ed714/content.

[32] Convention on Biological Diversity (2011). Nagoya Protocol on Access to Genetic Resources and the Fair and Equitable Sharing of Benefits Arising from Their Utilization to the Convention on Biological Diversity. https://www.cbd.int/abs/doc/protocol/nagoya-protocol-en.pdf.

[33] World Health Organization (2023). Genetic and Antigenic Characteristics of Zoonotic Influenza a Viruses and Development of Candidate Vaccine Viruses for Pandemic Preparedness. https://cdn.who.int/media/docs/default-source/influenza/who-influenza-recommendations/vcm-northern-hemisphere-recommendation-2023-2024/20230224_zoonotic_recommendations.pdf?sfvrsn=38c739fa_4.

[34] World Health Organization (2025). Summary of Status of Development and Availability of A(H5) non–A(H5N1) Candidate Vaccine Viruses and Potency Testing Reagents. https://cdn.who.int/media/docs/default-source/influenza/cvvs/cvv-zoonotic-northern-hemisphere-2025-2026/h5-non-h5n1_cvv_-20250228_20250813.pdf?sfvrsn=273530c8_7.

[35] World Health Organization (2025). Summary of Status of Development and Availability of A(H5N1) Candidate Vaccine Viruses and Potency Testing Reagents. https://cdn.who.int/media/docs/default-source/influenza/cvvs/cvv-zoonotic-northern-hemisphere-2025-2026/h5n1_summary_a_h5n1_cvv_20250228_rev20250813.pdf?sfvrsn=9c99e0a4_8.

[36] Gao F., Wang Q., Qiu C., Luo J., Li X. (2024). Pandemic preparedness of effective vaccines for the outbreak of newly H5N1 highly pathogenic avian influenza virus. Virol. Sin..

[37] Davis T. (2024). Highly Pathogenic Avian Influenza A(H5Nx) Virus Surveillance and Characterization in the United States and Globally and Recommendations for Candidate Vaccine Virus Development. https://www.fda.gov/media/182596/download.

[38] European Medicines Agency (2023). Assessment Report: Zoonotic Influenza Vaccine Seqirus. https://www.ema.europa.eu/en/documents/assessment-report/zoonotic-influenza-vaccine-seqirus-epar-public-assessment-report_en.pdf.

[39] World Health Organization (2018). Antigenic and Genetic Characteristics of Zoonotic Influenza Viruses and Development of Candidate Vaccine Viruses for Pandemic Preparedness. https://cdn.who.int/media/docs/default-source/influenza/who-influenza-recommendations/vcm-northern-hemisphere-recommendation-2018-2019/201802-zoonotic-vaccinevirusupdate.pdf.

[40] Kareinen L., Tammiranta N., Kauppinen A., Zecchin B., Pastori A., Monne I., Terregino C., Giussani E., Kaarto R., Karkamo V. (2024). Highly pathogenic avian influenza A(H5N1) virus infections on fur farms connected to mass mortalities of black-headed gulls, Finland, July to October 2023. Eurosurveillance.

[41] Domańska-Blicharz K., Świętoń E., Świątalska A., Monne I., Fusaro A., Tarasiuk K., Wyrostek K., Styś-Fijoł N., Giza A., Pietruk M. (2023). Outbreak of highly pathogenic avian influenza A(H5N1) clade 2.3.4.4b virus in cats, Poland, June to July 2023. Eurosurveillance.

[42] Agüero M., Monne I., Sánchez A., Zecchin B., Fusaro A., Ruano M.J., Arrojo M.d.V., Fernández-Antonio R., Souto A.M., Tordable P. (2023). Highly pathogenic avian influenza A(H5N1) virus infection in farmed minks, Spain, October 2022. Eurosurveillance.

[43] Maemura T., Guan L., Gu C., Eisfeld A., Biswas A., Halfmann P., Neumann G., Kawaoka Y. (2023). Characterization of highly pathogenic clade 2.3.4.4b H5N1 mink influenza viruses. EBioMedicine.

[44] Mirolo M., Pohlmann A., Ahrens A.K., Kühl B., Rubio-Garcìa A., Kramer K., Meinfelder U., Rosenberger T., Morito H.L., Beer M. (2023). Highly pathogenic avian influenza A virus (HPAIV) H5N1 infection in two European grey seals (*Halichoerus grypus*) with encephalitis. Emerg. Microbes Infect..

[45] World Health Organization (2025). Global Influenza Programme. https://www.who.int/teams/global-influenza-programme/vaccines/who-recommendations.

[46] Banzhoff A., Haertel S., Praus M. (2011). Passive surveillance of adverse events of an MF59-adjuvanted H1N1v vaccine during the pandemic mass vaccinations. Hum. Vaccines.

[47] Jelinek T., Schwarz T.F., Reisinger E., Malfertheiner P., Versage E., Van Twuijver E., Hohenboken M. (2024). Safety, Tolerability, and Immunogenicity of aH5N1 Vaccine in Adults with and without Underlying Medical Conditions. Vaccines.

[48] Malfertheiner P., Versage E., Van Twuijver E., Rizzardini G., Hohenboken M. (2025). Safety, Tolerability, and Immunogenicity of aH5N1 Vaccine in Adults with and Without Underlying Immunosuppressive Conditions. Vaccines.

[49] ClinicalTrials.gov (2025). BARDA BP-I-23-001 H5 Influenza. https://clinicaltrials.gov/study/NCT06560151.

[50] CSL Sequirus (2022). CSL Seqirus Announces U.S. Government Award to Manufacture and Clinically Assess Influenza A(H5N8) Pre-Pandemic Vaccine. https://www.cslseqirus.us/news/csl-seqirus-us-government-award-to-manufacture-and-assess-influenza-a-h5n8-pre-pandemic-vaccine.

[51] Belser J.A., Katz J.M., Tumpey T.M. (2011). The ferret as a model organism to study influenza A virus infection. Dis. Model. Mech..

[52] Albrecht R.A., Liu W.-C., Sant A.J., Tompkins S.M., Pekosz A., Meliopoulos V., Cherry S., Thomas P.G., Schultz-Cherry S. (2018). Moving Forward: Recent Developments for the Ferret Biomedical Research Model. mBio.

[53] Dunning A.J., DiazGranados C.A., Voloshen T., Hu B., Landolfi V.A., Talbot H.K. (2016). Correlates of Protection against Influenza in the Elderly: Results from an Influenza Vaccine Efficacy Trial. Clin. Vaccine Immunol..

[54] Coudeville L., Bailleux F., Riche B., Megas F., Andre P., Ecochard R. (2010). Relationship between haemagglutination-inhibiting antibody titres and clinical protection against influenza: Development and application of a bayesian random-effects model. BMC Med. Res. Methodol..

[55] Hobson D., Curry R.L., Beare A.S., Ward-Gardner A. (1972). The role of serum haemagglutination-inhibiting antibody in protection against challenge infection with influenza A2 and B viruses. Epidemiol. Infect..

[56] Gilbert P.B., Fong Y., Juraska M., Carpp L.N., Monto A.S., Martin E.T., Petrie J.G. (2019). HAI and NAI titer correlates of inactivated and live attenuated influenza vaccine efficacy. BMC Infect. Dis..

[57] Danier J., Callegaro A., Soni J., Carmona A., Kosalaraska P., Rivera L., Friel D., Pu W., Vantomme V., Dbaibo G. (2021). Association Between Hemagglutination Inhibition Antibody Titers and Protection Against Reverse-Transcription Polymerase Chain Reaction–Confirmed Influenza Illness in Children 6–35 Months of Age: Statistical Evaluation of a Correlate of Protection. Open Forum Infect. Dis..

[58] Wong S.-S., Duan S., DeBeauchamp J., Zanin M., Kercher L., Sonnberg S., Fabrizio T., Jeevan T., Crumpton J.-C., Oshansky C. (2017). The immune correlates of protection for an avian influenza H5N1 vaccine in the ferret model using oil-in-water adjuvants. Sci. Rep..

[59] Suguitan A.L., McAuliffe J., Mills K.L., Jin H., Duke G., Lu B., Luke C.J., Murphy B., Swayne D.E., Kemble G. (2006). Live, Attenuated Influenza A H5N1 Candidate Vaccines Provide Broad Cross-Protection in Mice and Ferrets. PLoS Med..

[60] European Medicines Agency (2016). Guideline on Influenza Vaccines (Non-Clinical and Clinical Module). https://www.ema.europa.eu/en/documents/scientific-guideline/influenza-vaccines-non-clinical-and-clinical-module_en.pdf.

[61] Belser J.A., Eckert A.M., Huynh T., Gary J.M., Ritter J.M., Tumpey T.M., Maines T.R. (2020). A Guide for the Use of the Ferret Model for Influenza Virus Infection. Am. J. Pathol..

[62] U.S. Department of Health and Human Services Food and Drug Administration (2007). Guidance for Industry Clinical Data Needed to Support the Licensure of Pandemic Influenza Vaccines. https://www.fda.gov/files/vaccines,%20blood%20&%20biologics/published/Guidance-for-Industry--Clinical-Data-Needed-to-Support-the-Licensure-of-Pandemic-Influenza-Vaccines.pdf.

[63] Segovia K., Rathnasinghe R., Patton C., Kwon B., Longstaff R.A., Hofmann D., Banger K.K., Xu H., Lacey M., Settembre E. (2026). MF59-Adjuvanted A/Astrakhan Influenza Vaccine Induces Cross-Neutralizing H5N1 Antibodies in Ferrets Against Circulating Clade 2.3.4.4b Viruses.

[64] Thimmiraju S.R., Kimata J.T., Pollet J. (2024). Pseudoviruses, a safer toolbox for vaccine development against enveloped viruses. Expert Rev. Vaccines.

[65] Liedes O., Reinholm A., Ekström N., Haveri A., Solastie A., Vara S., Rijnink W.F., Bestebroer T.M., Richard M., de Vries R.D. (2025). Influenza A(H5N8) vaccine induces humoral and cell-mediated immunity against highly pathogenic avian influenza clade 2.3.4.4b A(H5N1) viruses in at-risk individuals. Nat. Microbiol..

[66] Wadey C., Rockman S. (2024). Analysing the Potency of a Seasonal Influenza Vaccine Using Reference Antisera from Heterologous Strains. Vaccines.

[67] European Commission (2024). Commission Secures Access to 665,000 Doses of Zoonotic Influenza Vaccines. https://health.ec.europa.eu/latest-updates/commission-secures-access-665000-doses-zoonotic-influenza-vaccines-2024-06-11_en.

[68] Sparrow E., Wood J.G., Chadwick C., Newall A.T., Torvaldsen S., Moen A., Torelli G. (2021). Global production capacity of seasonal and pandemic influenza vaccines in 2019. Vaccine.

[69] Minor P.D. (2015). Assaying the Potency of Influenza Vaccines. Vaccines.

[70] European Court of Auditors (2022). EU COVID-19 Vaccine Procurement. https://www.eca.europa.eu/Lists/ECADocuments/SR22_19/SR_EU_COVID_vaccine_procurement_EN.pdf.

[71] UK Health Security Agency (2024). UK Secures H5 Influenza Vaccine to Boost Pandemic Preparedness. https://www.gov.uk/government/news/uk-secures-h5-influenza-vaccine-to-boost-pandemic-preparedness.

[72] European Medicines Agency (2025). Zoonotic Influenza Vaccine Seqirus—Procedural Steps Taken and Scientific Information After the Authorisation. https://www.ema.europa.eu/en/documents/procedural-steps-after/zoonotic-influenza-vaccine-seqirus-epar-procedural-steps-taken-scientific-information-after-authorisation-archive_en.pdf?utm.

[73] CSL Seqirus (2026). CSL Seqirus Announces Third U.S. Government Award in Relation to Influenza A(H5N8) Candidate Vaccine. https://www.prnewswire.com/news-releases/csl-seqirus-announces-third-us-government-award-in-relation-to-influenza-ah5n8-candidate-vaccine-301911153.html.

